# Supplementary material for: Discovering type I cis-AT polyketides through computational mass spectrometry and genome mining with Seq2PKS
Source: Nat Commun. 2024 Jun 25;15:5356. doi: 10.1038/s41467-024-49587-1 (PMC11199612; doi:10.1038/s41467-024-49587-1)
Supplement: Supplementary file 1 — Supplementary Information [file 41467_2024_49587_MOESM1_ESM.pdf]

Supplementary Information for Discovering type I cis-AT polyketides through computational mass spectrometry and genome mining with Seq2PKS

Donghui Yan *[1], Muqing Zhou *[1], Abhinav Adduri *[1], Yihao Zhuang[2], Mustafa Guler[1], Sitong Liu[1], Hyonyoung Shin[1], Torin Kovach[1], Gloria Oh[1], Xiao Liu[1], Yuting Deng[1], Xiaofeng Wang[3], Liu Cao[1], David H. Sherman[3,4], Pamela J. Schultz[2,4], Roland D. Kersten[3], Jason A. Clement[5], Ashootosh Tripathi †[2,3,4], Bahar Behsaz †[1,6], and Hosein Mohimani †[1]

[1]Computational Biology Department, School of Computer Science, Carnegie Mellon University, PA 15213, USA

[2]Natural Products Discovery Core, University of Michigan, Ann Arbor, MI 48109, USA

[3]Department of Medicinal Chemistry, University of Michigan, Ann Arbor, MI 48109, USA

[4]Life Sciences Institute, University of Michigan, Ann Arbor, MI 48109, USA

[5]Baruch S. Blumberg Institute, 3805 Old Easton Road, Doylestown, PA 18902, USA

[6]Chemia Biosciences, Pittsburgh, PA 15217, USA

* These authors contributed equally to this work.

† Corresponding authors: ashtri@umich.edu, bahaar@gmail.com, hoseinm@andrew.cmu.edu

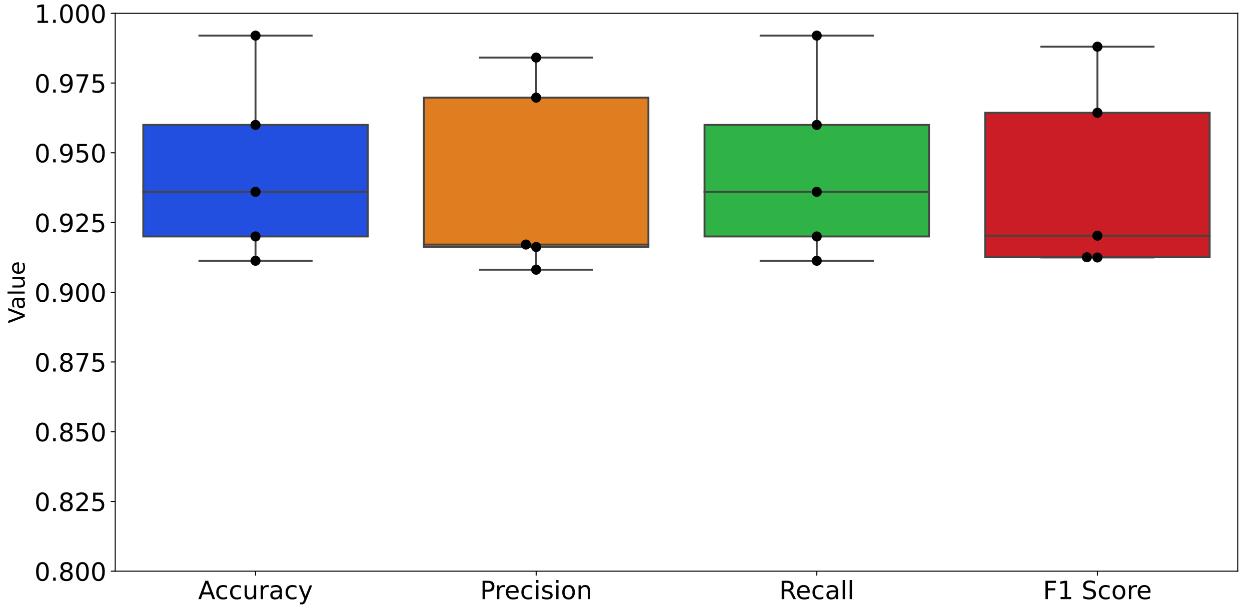

Supplementary Figure 1: Evaluation metrics for the extra-tree algorithm in predicting AT domain specificity during five-fold cross-validation. Error bars represent the standard deviation.

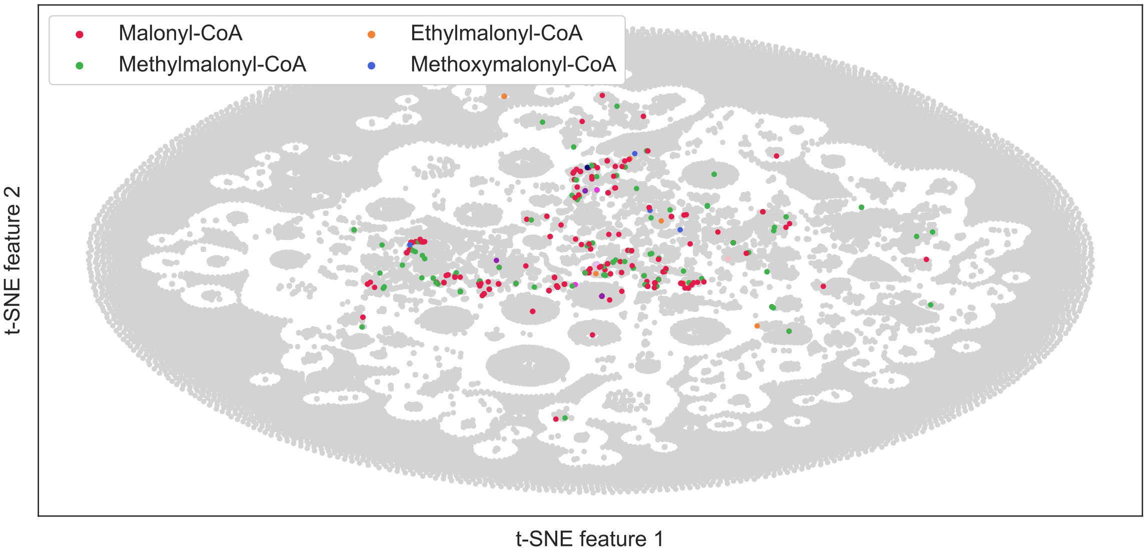

Supplementary Figure 2: t-SNE plot of signatures from all 175,201 AT-domains extracted from modular polyketide microbial genome sequences from RefSeq database. Each point corresponds to a domain. The gray points correspond to domains with unknown specificities, while the rest correspond to known specificities. t-SNE is performed based on the normalized Euclidean distance of the domains in the feature space. Part of the space remains completely unexplored (gray zone), and it could correspond to novel specificities.

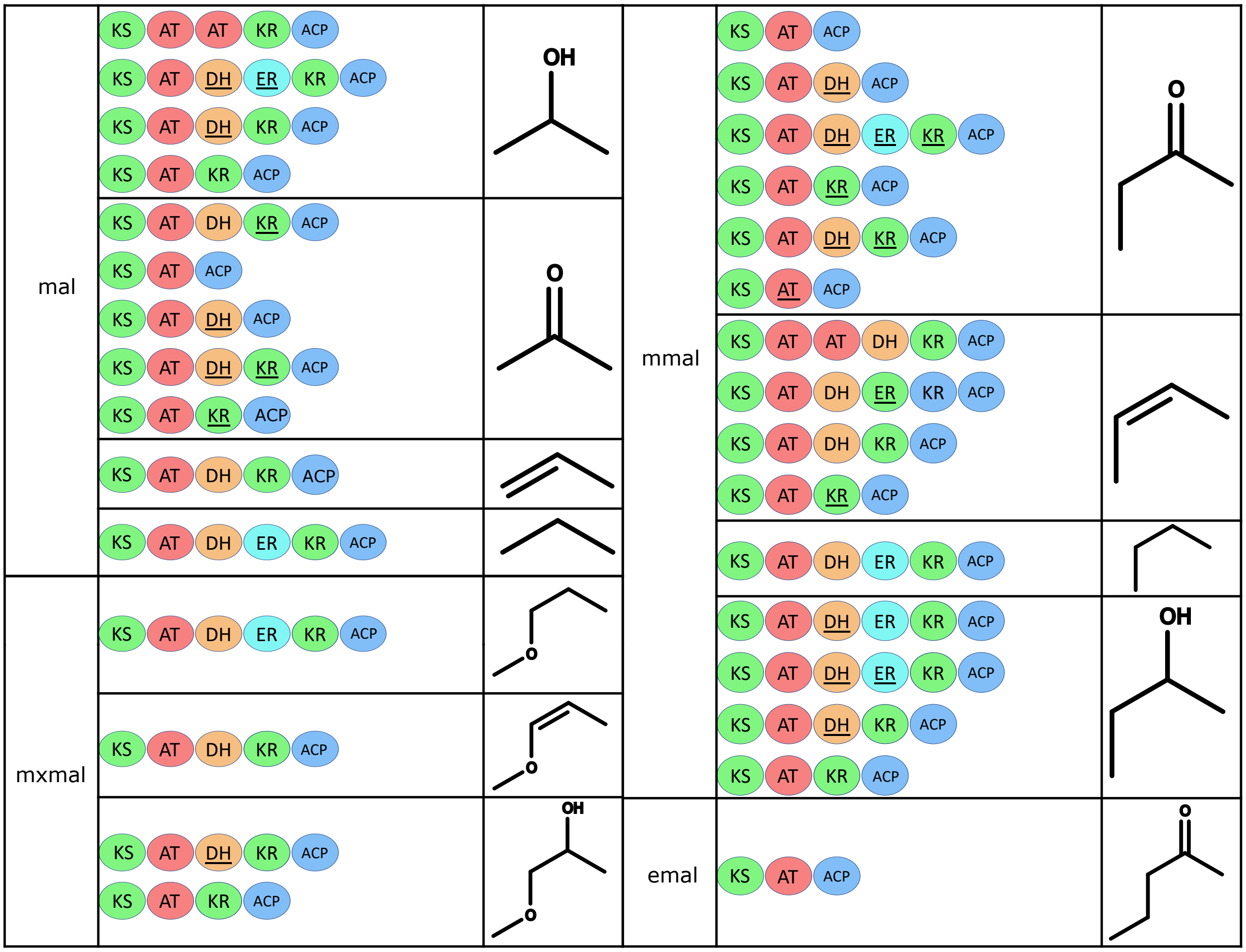

Supplementary Figure 3: 39 rules that govern the formation of mature substrates based on AT-domain specificity and the existence of other domains in Cis-AT modules. These rules are extracted from 191 Cis-AT modules with known specificity from 80 Cis-AT polyketides by literature mining. Inactive domains are labeled with an underline. AT: Acyltransferase; KS: ketosynthase; DH: dehydratase; KR: ketoreductase; ER: enoylreductase; ACP: acyl-acyl carrier protein. mal: malonyl-CoA; mmal: methylmalonyl-CoA; mxmal: methoxymalonyl-CoA; emal: ethylmalonyl-CoA.

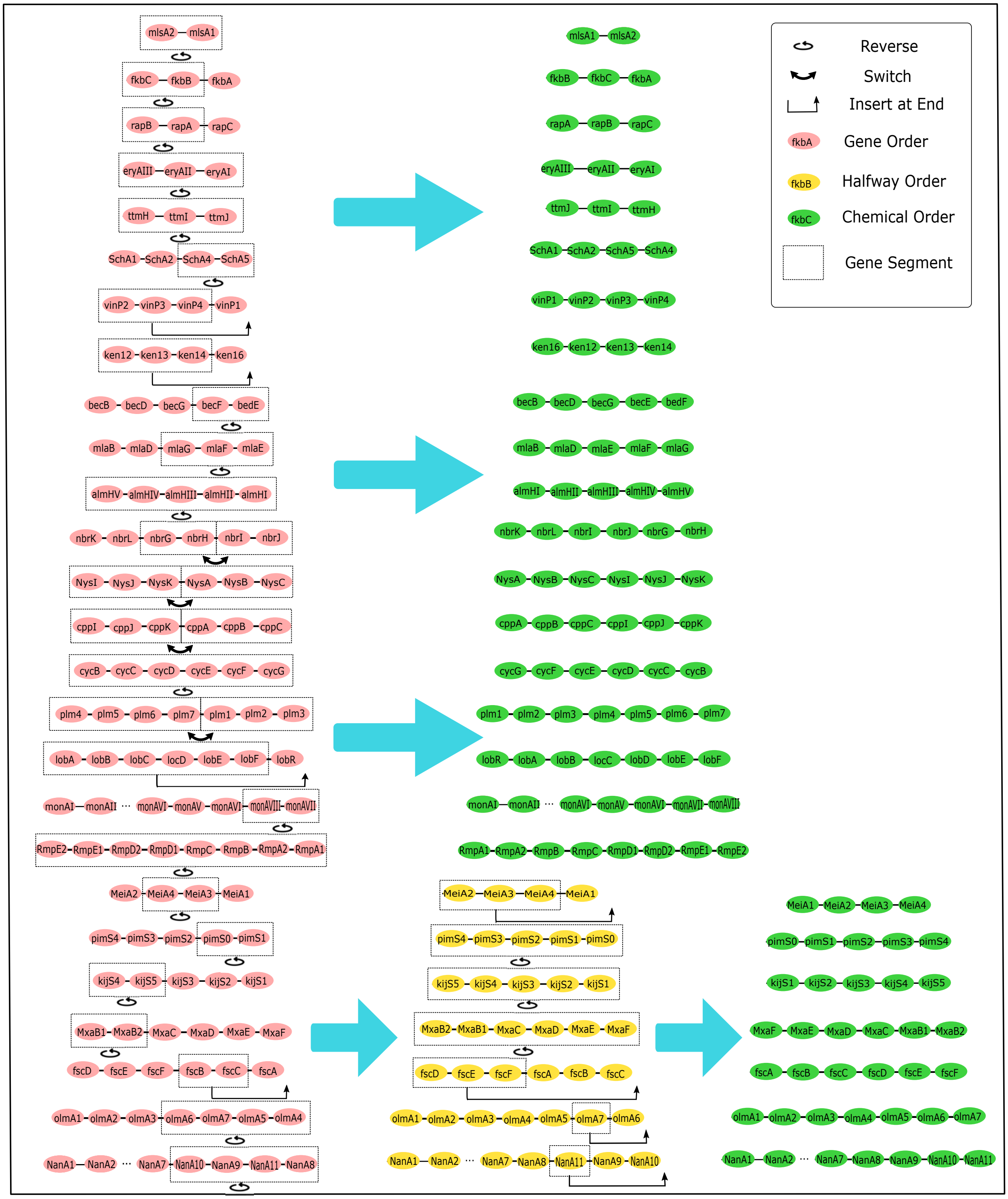

Supplementary Figure 4: Substrates reordering during core structure construction. The majority of the re-orderings can be explained by reversion, switching, and insertion.

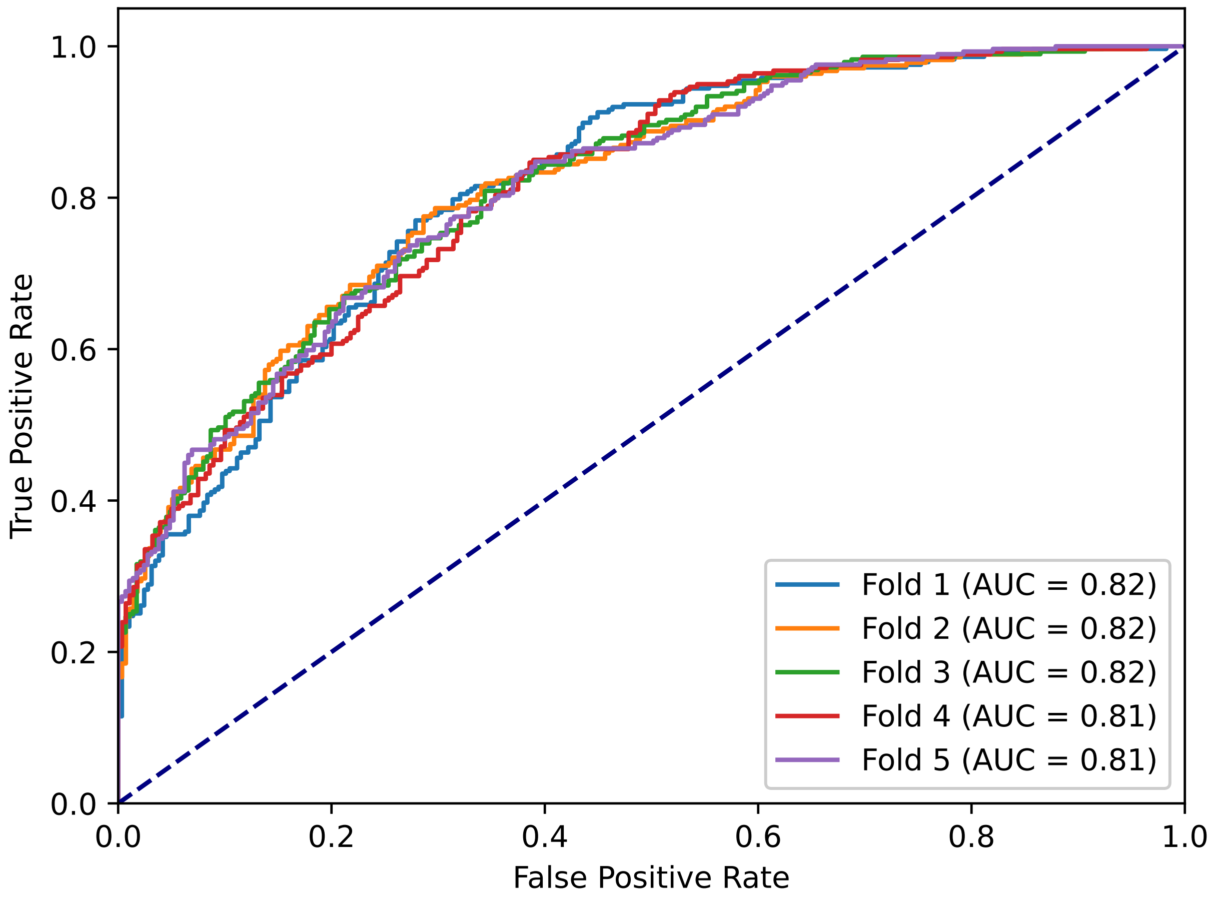

Supplementary Figure 5: ROC-AUC curve for PNN method during five-fold cross-validation process.

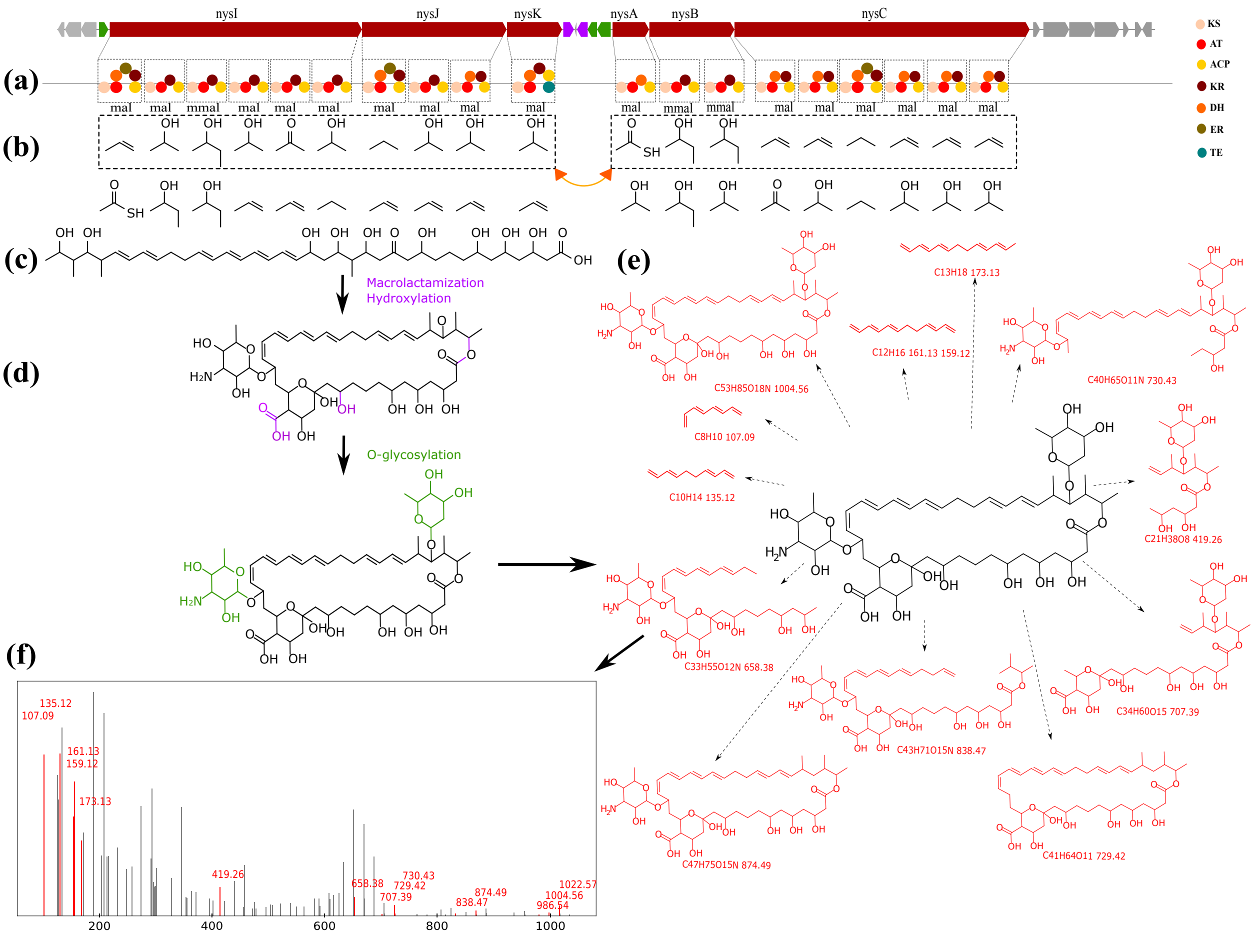

Supplementary Figure 6: Identification of nystatin by Seq2PKS. (a) Domains and modules in the biosynthetic genes are identified. (b) Substrate specificity for each AT domain is predicted. (c) Assembly order is predicted, and the core structure is constructed by using the predicted substrates for each module. (d) Corresponding modifications are applied to the core structure to form hypothetical molecules. (e) Fragments from the nystatin molecule (shown in the middle in black) that match a peak in mass spectra are highlighted. Fragments are generated by one or two rounds of fragmentation of the nystatin molecule. (f) The annotated spectrum of nystatin is shown in red.

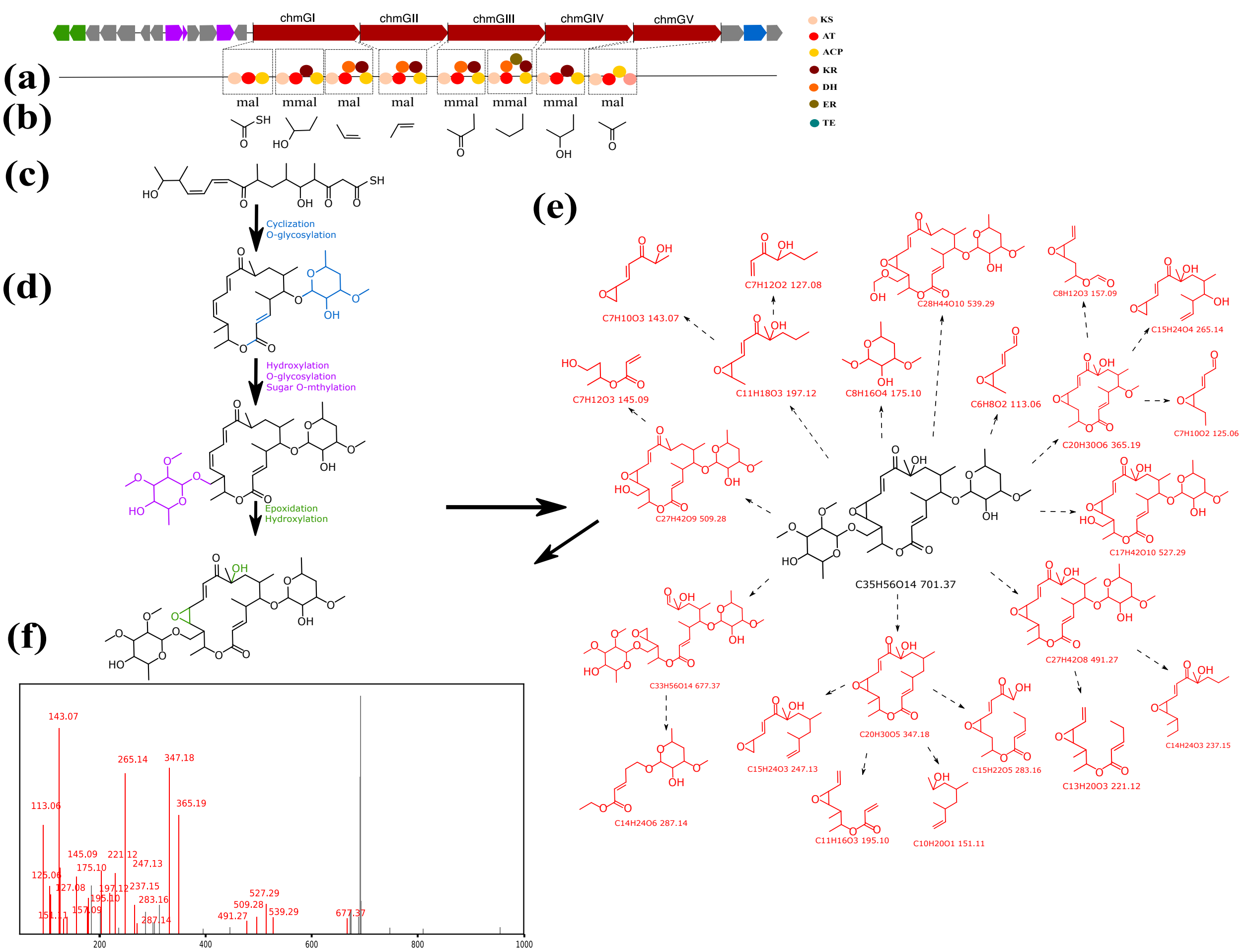

Supplementary Figure 7: Identification of chalcomycin by Seq2PKS. (a) Domains and modules in the biosynthetic genes are identified. (b) Substrate specificity for each AT domain is predicted. (c) Assembly order is predicted, and the core structure is constructed using the predicted substrates for each module. (d) Corresponding modifications are applied to the core structure to form hypothetical molecules. (e) Fragments from the chalcomycin molecule (shown in the middle in black) that match a peak in the mass spectra are highlighted. Fragments are generated by one or two rounds of fragmentation of the chalcomycin molecule. (f) The annotated spectrum of chalcomycin is shown in red.

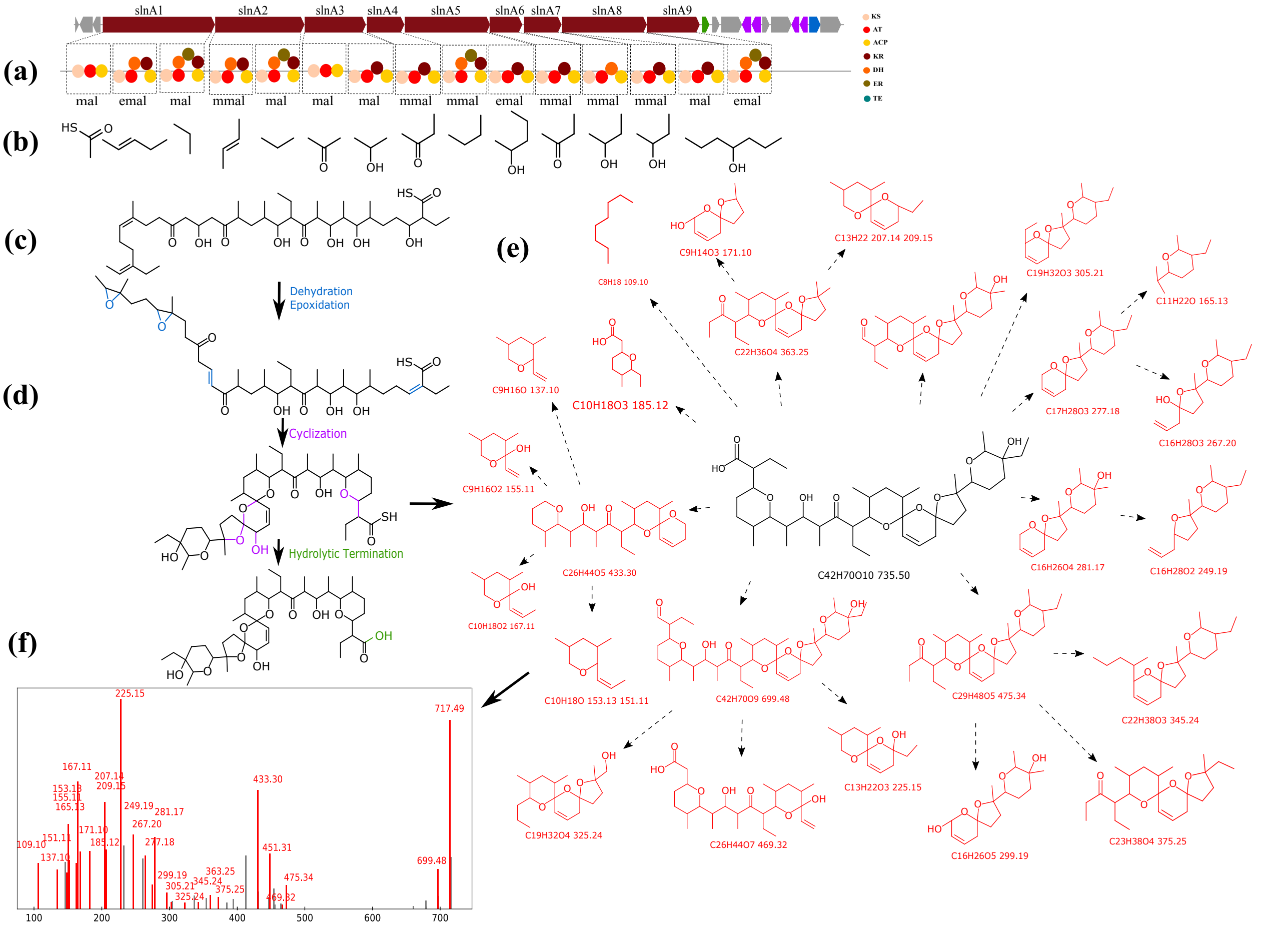

Supplementary Figure 8: Identification of salinomycin by Seq2PKS. (a) Domains and modules in the biosynthetic genes are identified. (b) Substrate specificity for each AT domain is predicted. (c) Assembly order is predicted, and the core structure is constructed by using the predicted substrates for each module. (d) Corresponding modifications are applied to the core structure to form hypothetical molecules. (e) Fragments from the salinomycin molecule (shown in the middle in black) that match a peak in mass spectra are highlighted. Fragments are generated by one or two rounds of fragmentation of the salinomycin molecule. (f) The annotated spectrum of salinomycin+ is shown in red.

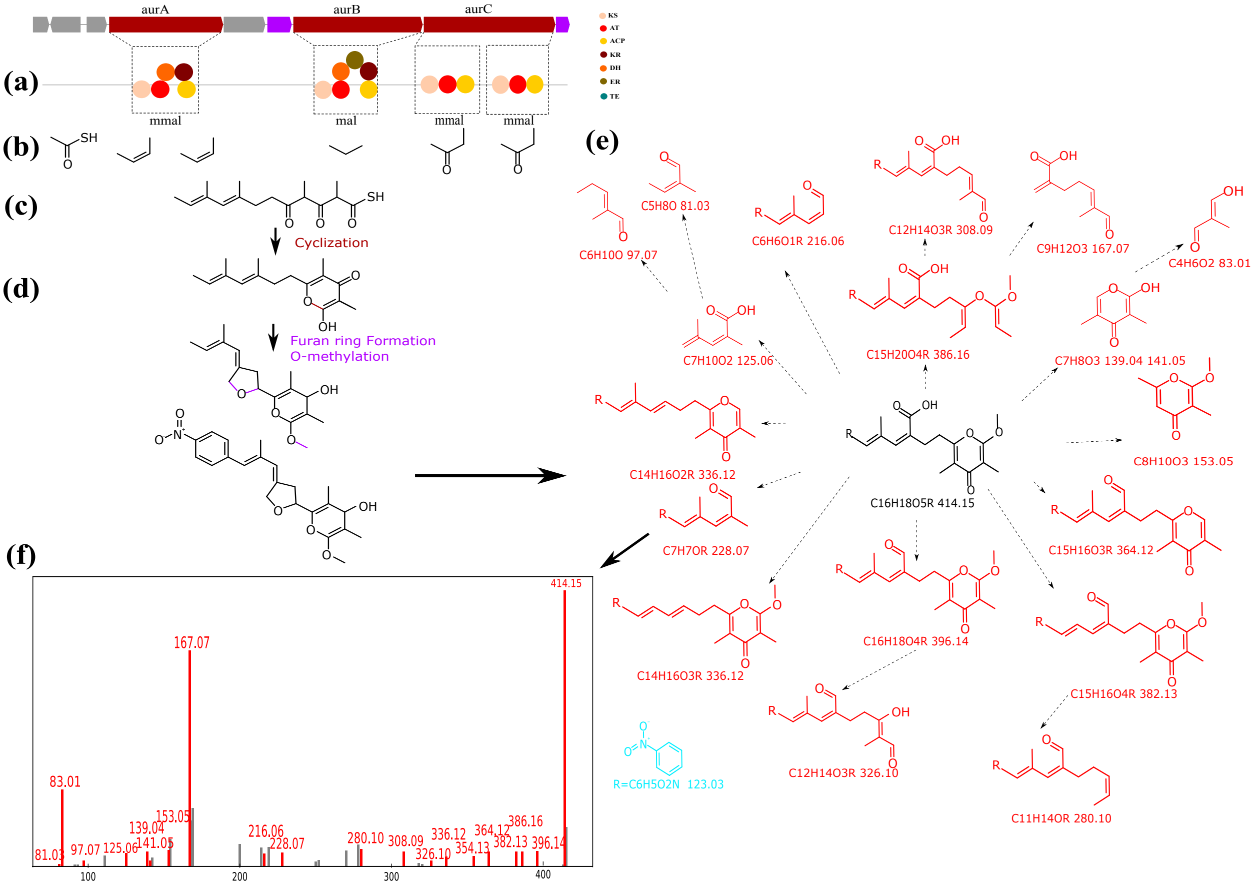

Supplementary Figure 9: Identification of aureothin by Seq2PKS. (a) Domains and modules in the biosynthetic genes are identified. (b) Substrate specificity for each AT domain is predicted. (c) Assembly order is predicted, and the core structure is constructed by using the predicted substrates for each module. (d) Corresponding modifications are applied to the core structure to form hypothetical molecules. (e) Fragments from the aureothin molecule (shown in the middle in black) that match a peak in mass spectra are highlighted. Fragments are generated by one or two rounds of fragmentation of the aureothin molecule. The starter unit is shown in blue. (f) The annotated spectrum of aureothin is shown in red.

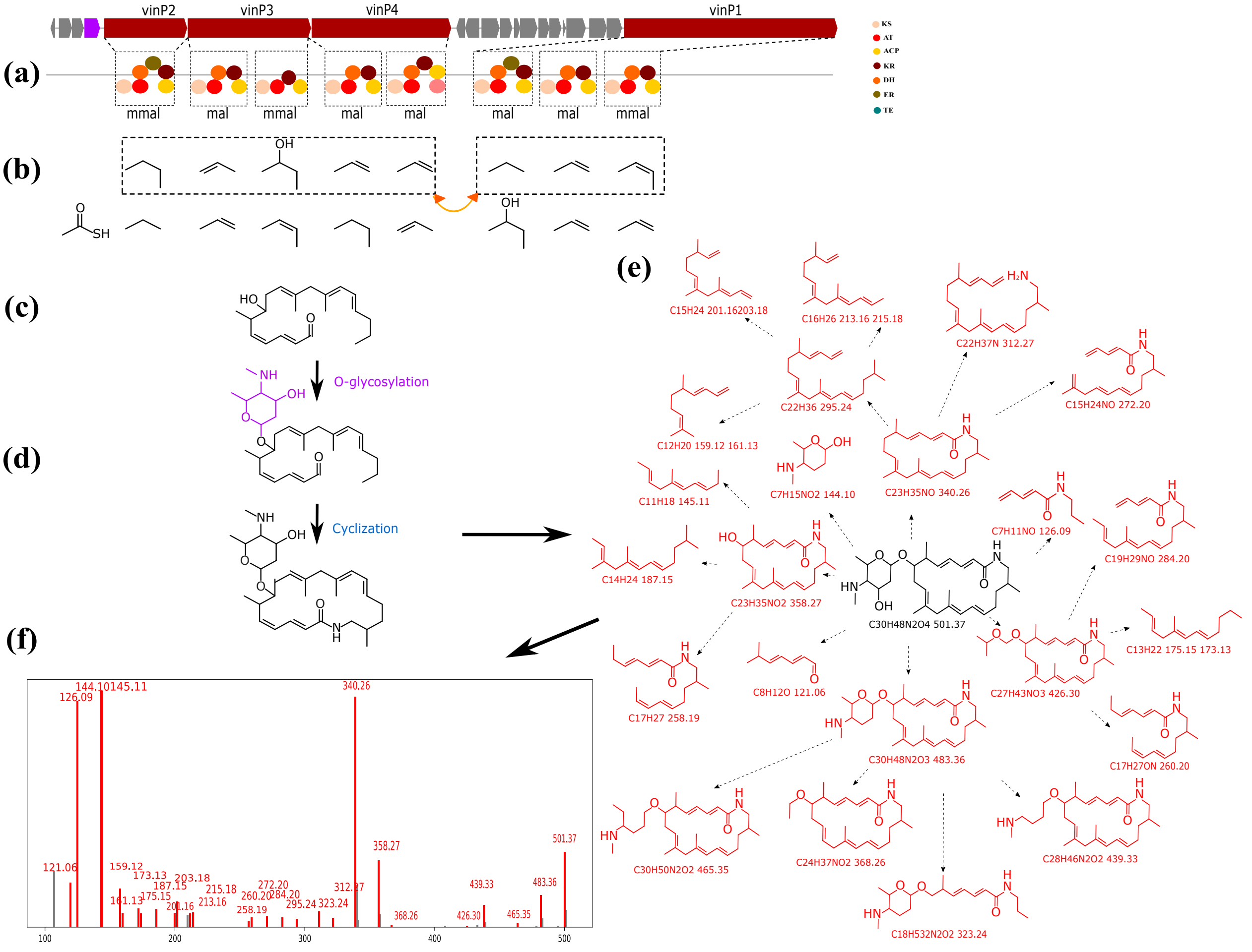

Supplementary Figure 10: Identification of viceinstatin by Seq2PKS. (a) Domains and modules in the biosynthetic genes are identified. (b) Substrate specificity for each AT domain is predicted. (c) Assembly order is predicted, and the core structure is constructed by using the predicted substrates for each module. (d) Corresponding modifications are applied to the core structure to form hypothetical molecules. (e) Fragments from the viceinstatin molecule (shown in the middle in black) that match a peak in mass spectra are highlighted. Fragments are generated by one or two rounds of fragmentation of the viceinstatin molecule. (f) The annotated spectrum of viceinstatin is shown in red.

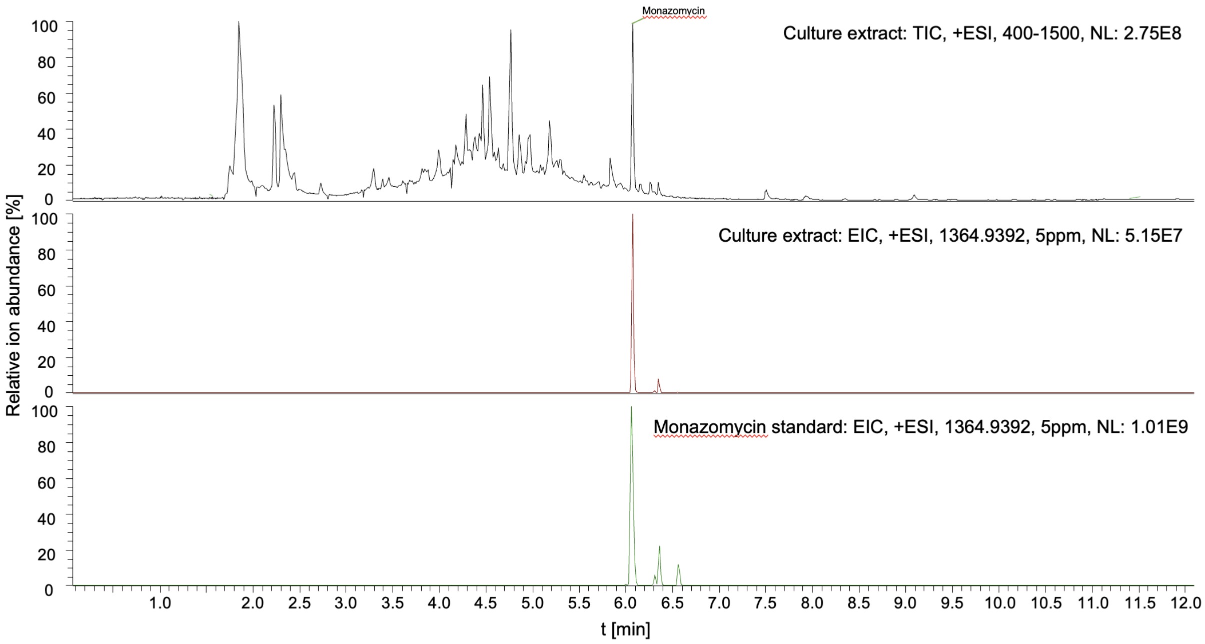

Supplementary Figure 11: LCMS-based comparison of monazomycin analyte from our cultured strain *Streptomyces cinnamoneus* NRRL B-24434 and an authentic standard provided by Santa Cruz Biotechnology.

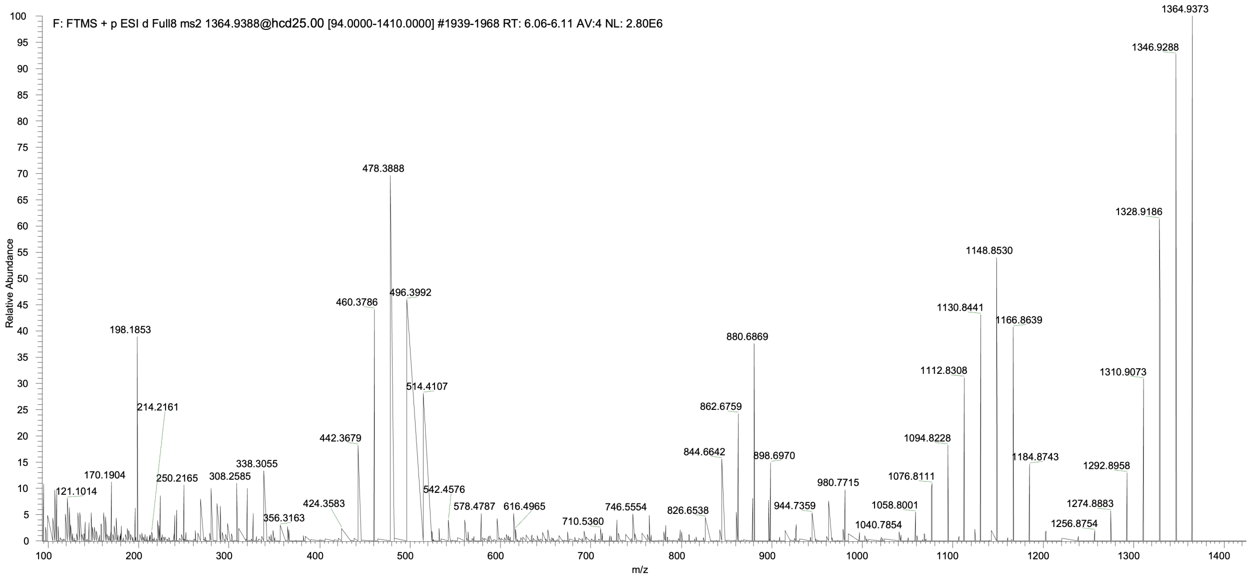

(a)

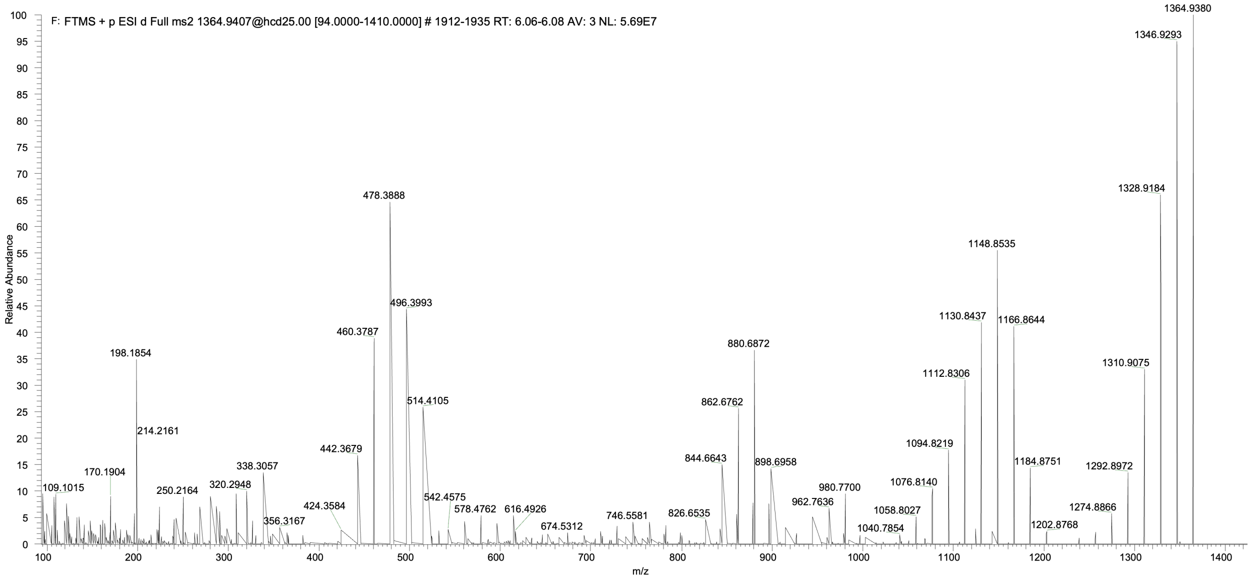

(b)

Supplementary Figure 12: Tandem mass spectra for monazomycin from (a) our cultured strain *Streptomyces cinnamoneus* NRRL B-24434 and (b) authentic standard provided by Santa Cruz Biotechnology.

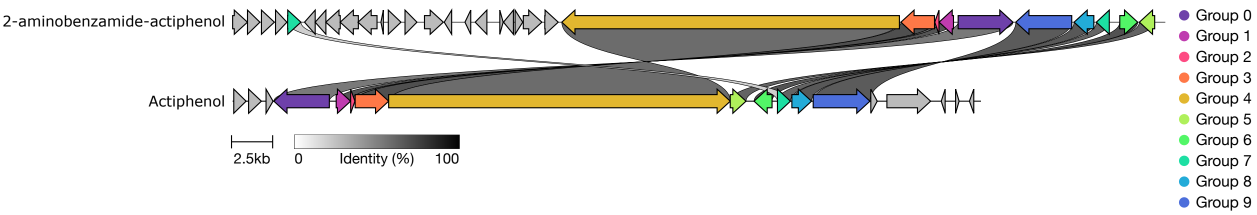

Supplementary Figure 13: Comparison of Biosynthetic Gene Clusters (BGCs) between the 2-aminobenzamide-actiphenol and actiphenol from the MIBiG database (BGC0000175).

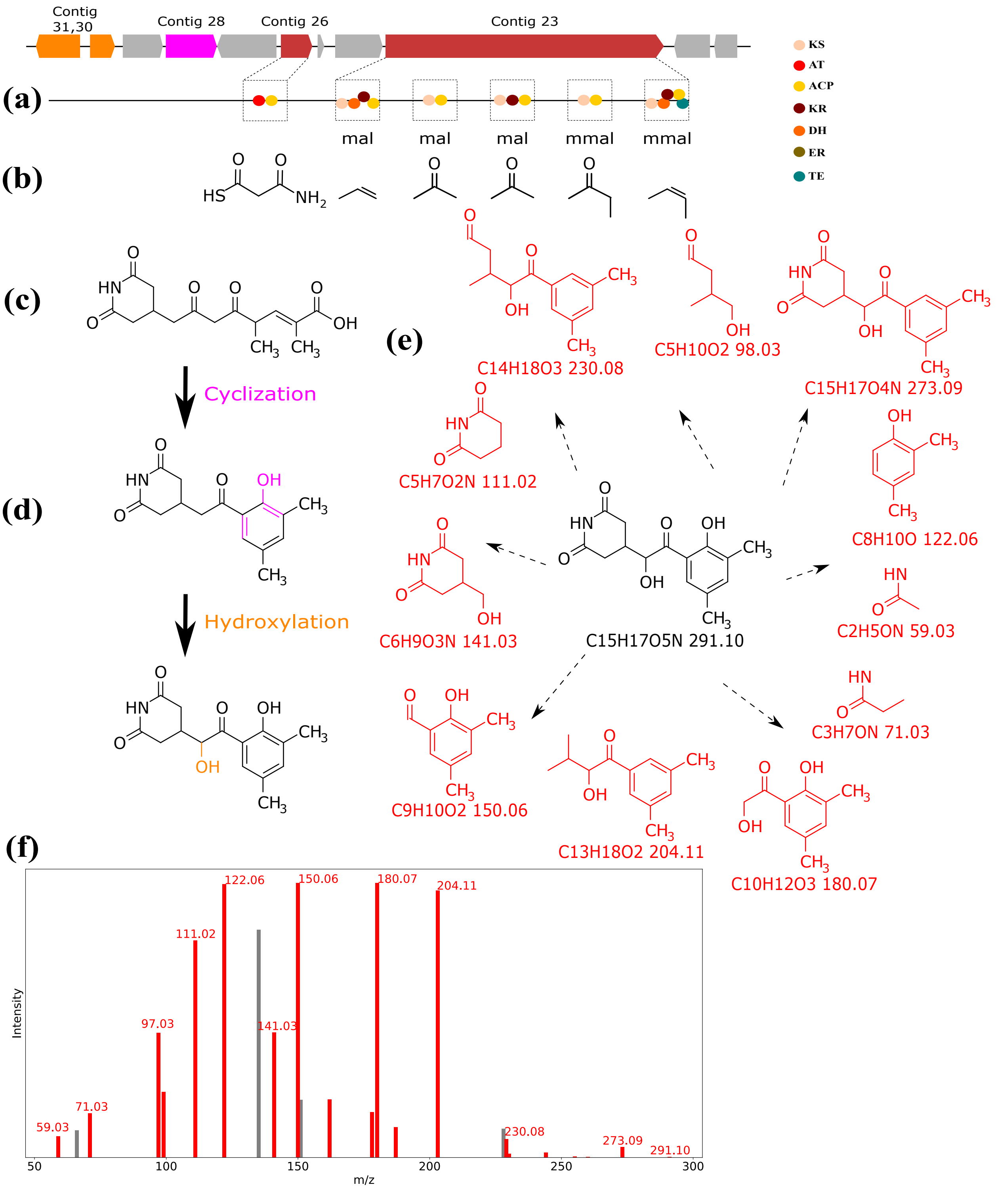

Supplementary Figure 14: Identification of Nong-Kang 101-G by Seq2PKS. (a) Domains and modules in the biosynthetic genes are identified. (b) Substrate specificity and mature substrate for each module are predicted. (c) Assembly order is predicted, and the core structure is constructed by connecting the predicted mature substrates for each module. (d) Cyclization and hydroxylation modifications are applied by two enzymes from the BGCs (shown in pink and orange, respectively). (e) Fragments from the hypothetical molecule (shown in black) that match a peak in mass spectra are highlighted (shown in red). Fragments are generated by one or two rounds of fragmentation of the hypothetical molecule. (f) The annotated spectrum of the hypothetical molecule is shown in red.

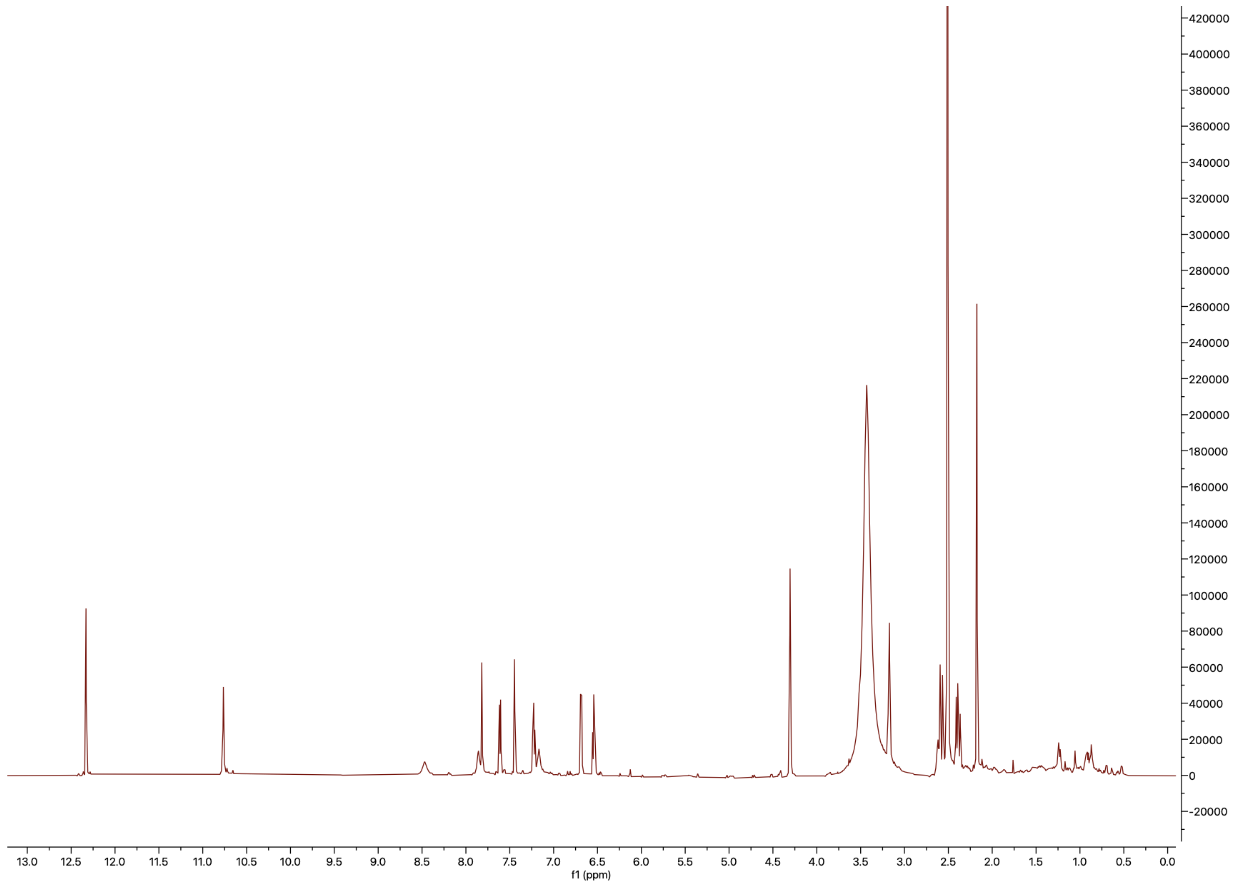

Supplementary Figure 15: ^{1}H spectrum of 2-aminobenzamide-actiphenol.

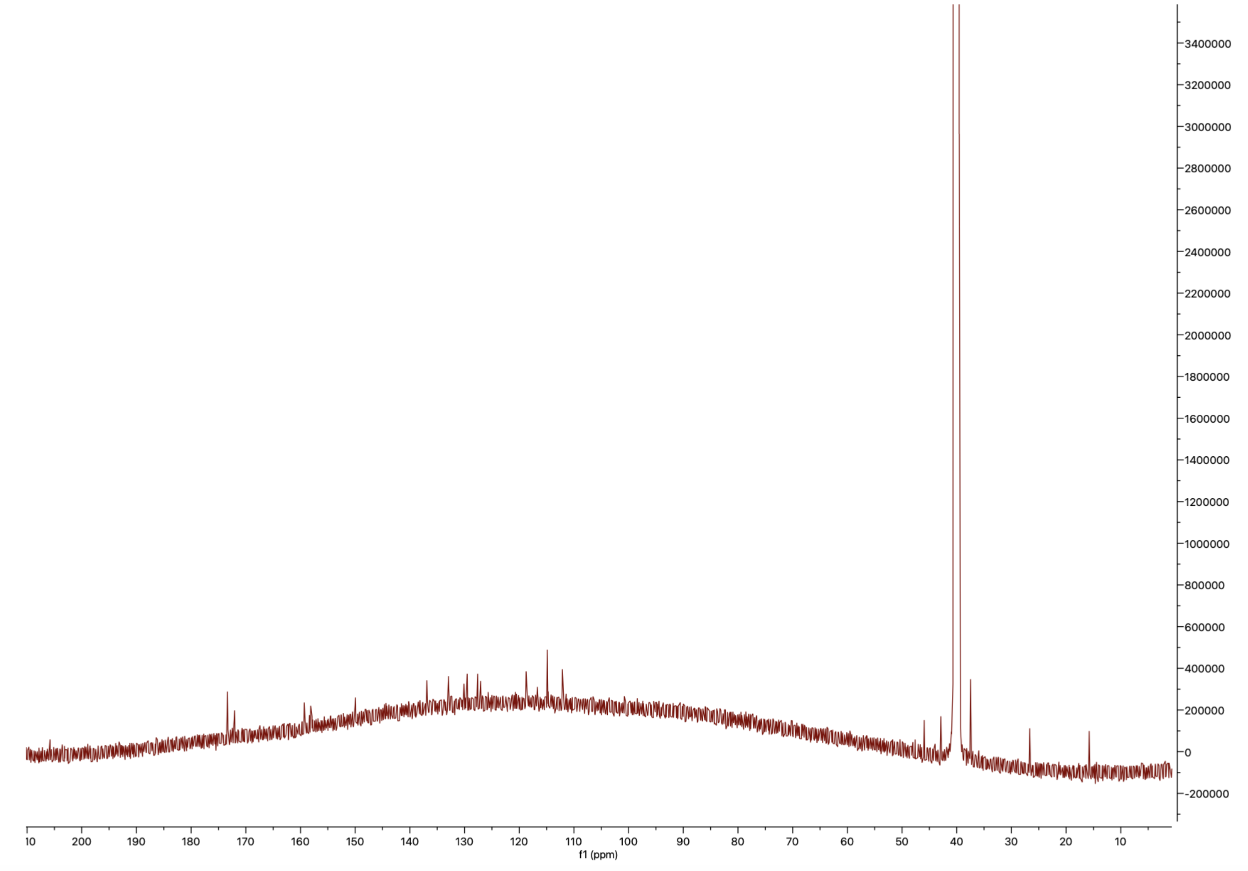

Supplementary Figure 16: ^{13}C spectrum of 2-aminobenzamide-actiphenol.

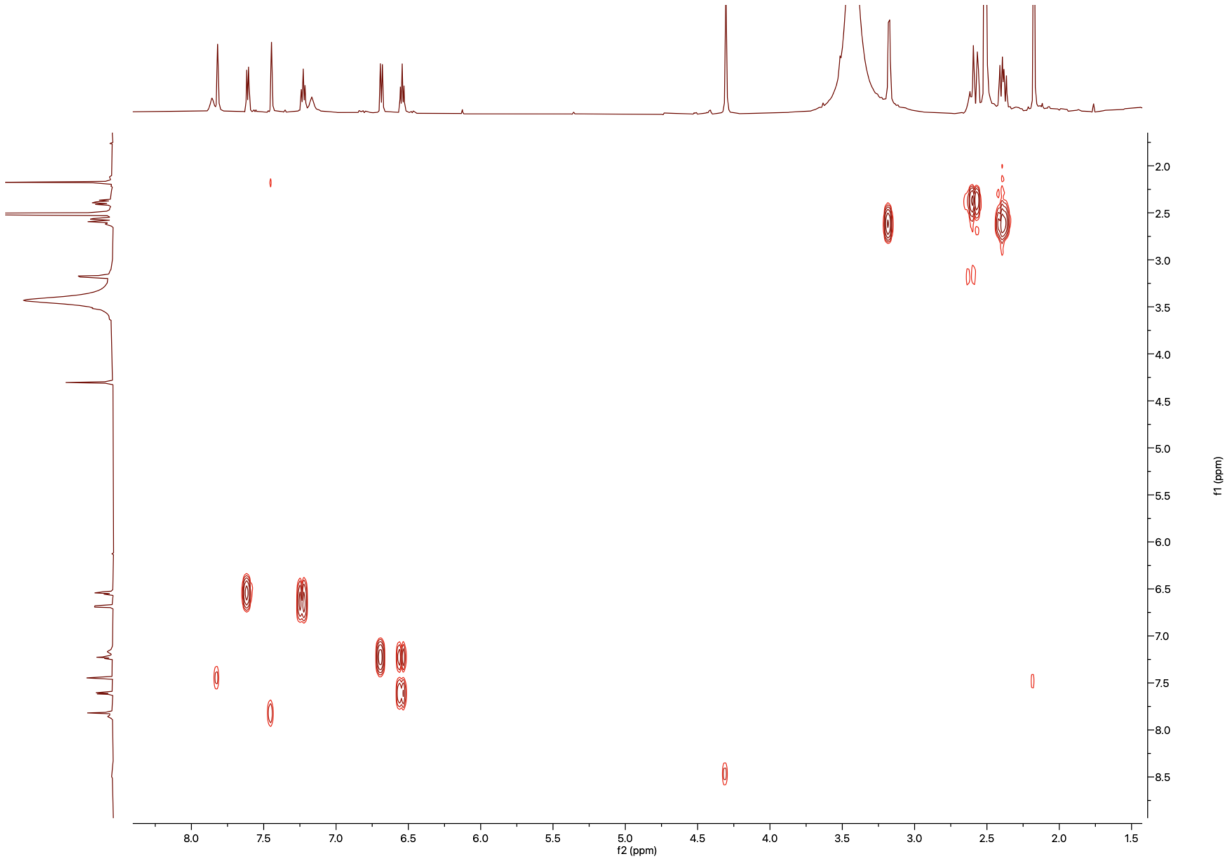

Supplementary Figure 17: COSY spectrum of 2-aminobenzamide-actiphenol.

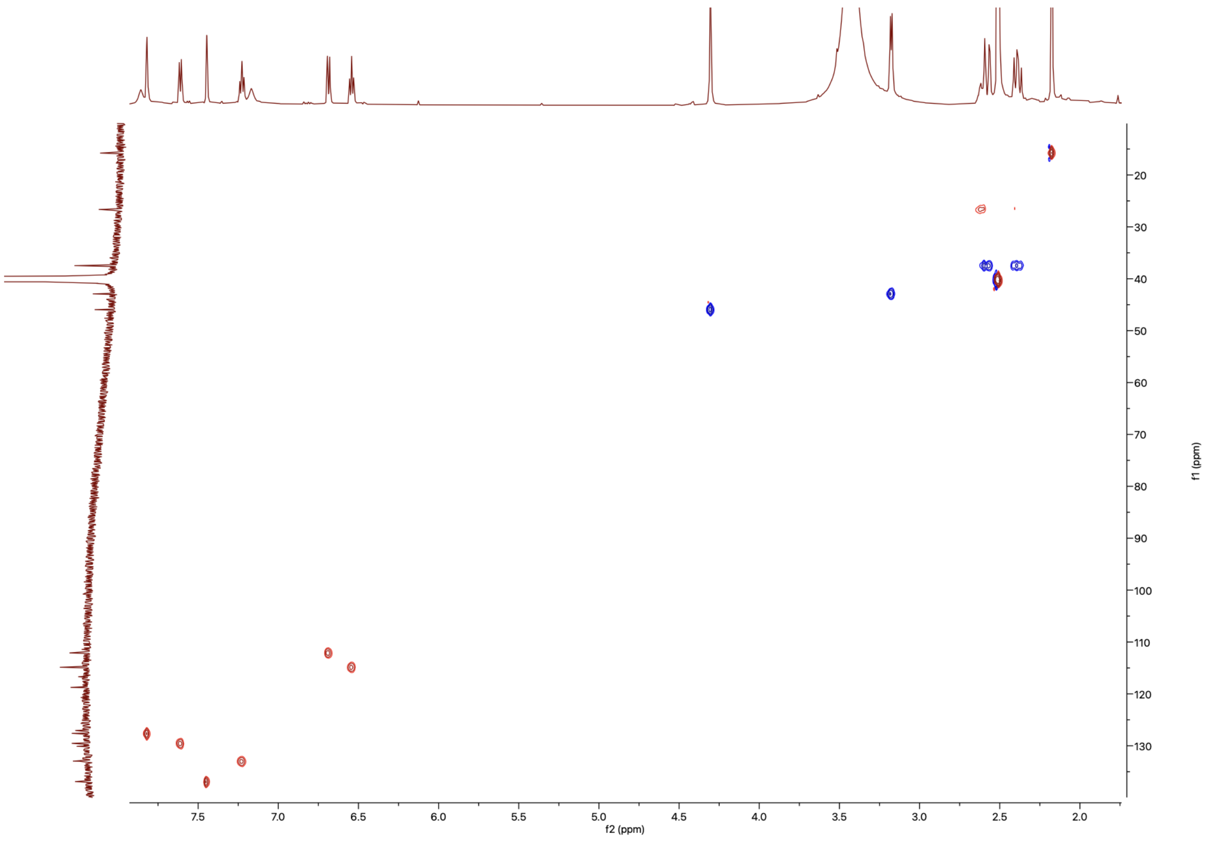

Supplementary Figure 18: HSQC spectrum of 2-aminobenzamide-actiphenol.

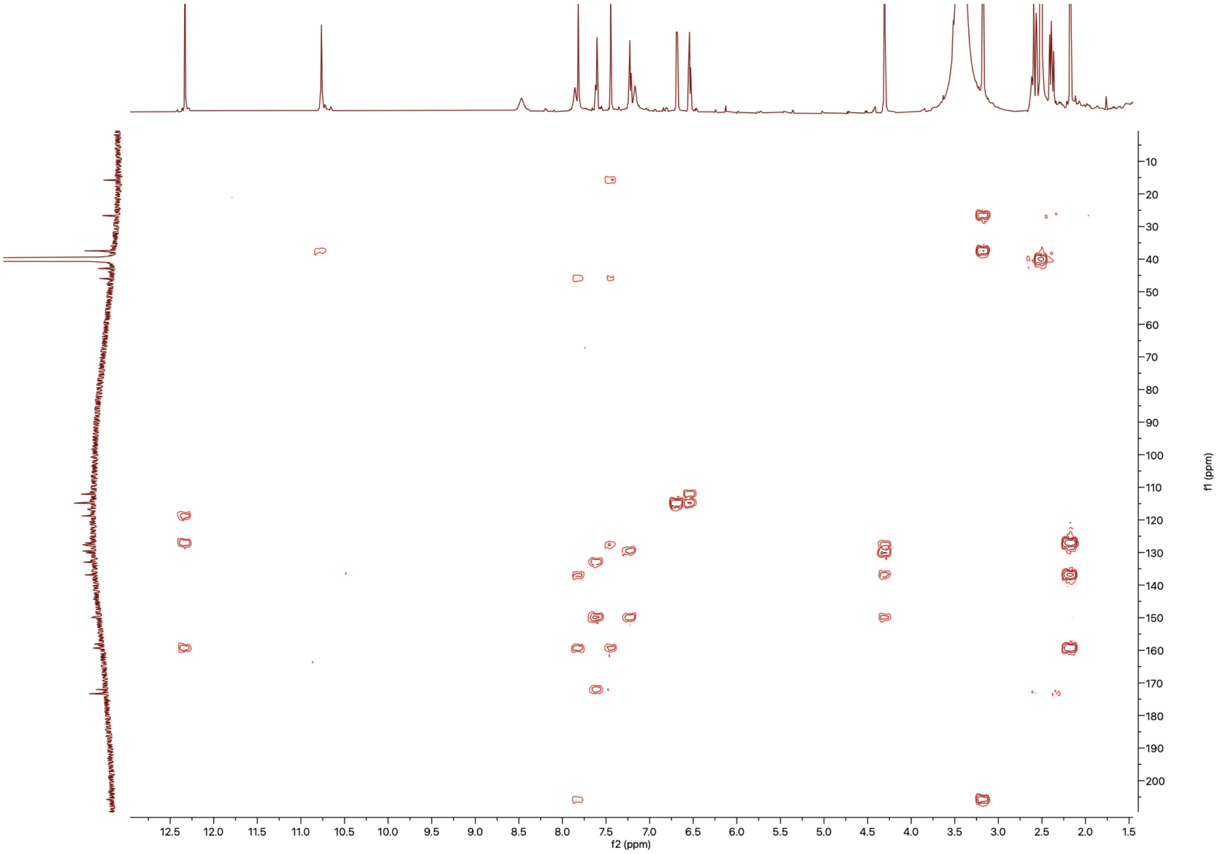

Supplementary Figure 19: ^{1}H-^{13}C HMBC spectrum of 2-aminobenzamide-actiphenol.

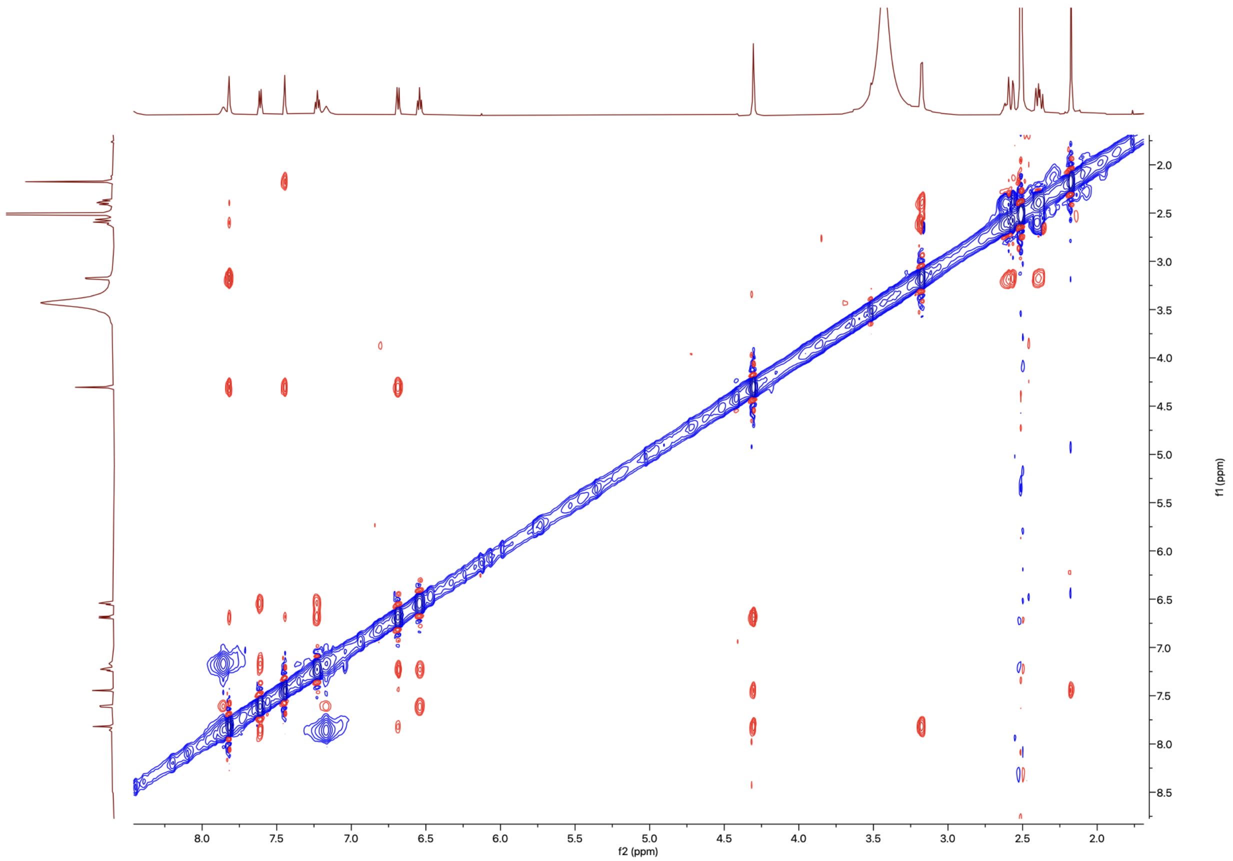

Supplementary Figure 20: ROESY spectrum of 2-aminobenzamide-actiphenol.

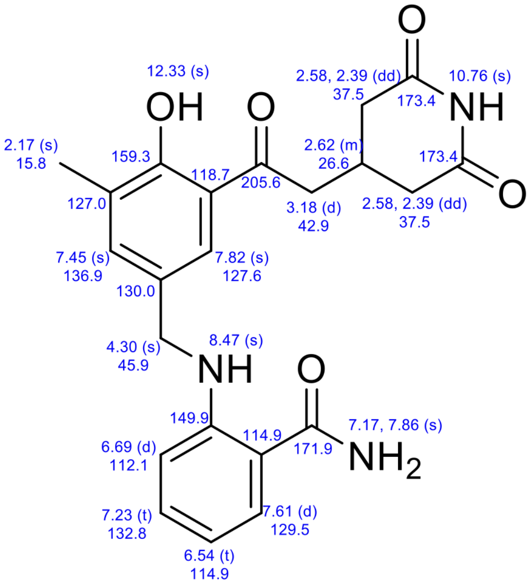

Chemical Formula: $C_{22}H_{23}N_3O_5$

Exact Mass: 409.16

Supplementary Figure 21: ^{1}H NMR Spectral Analysis showcasing the chemical shifts and multiplicity patterns for the identification of 2-aminobenzamide-actiphenol.

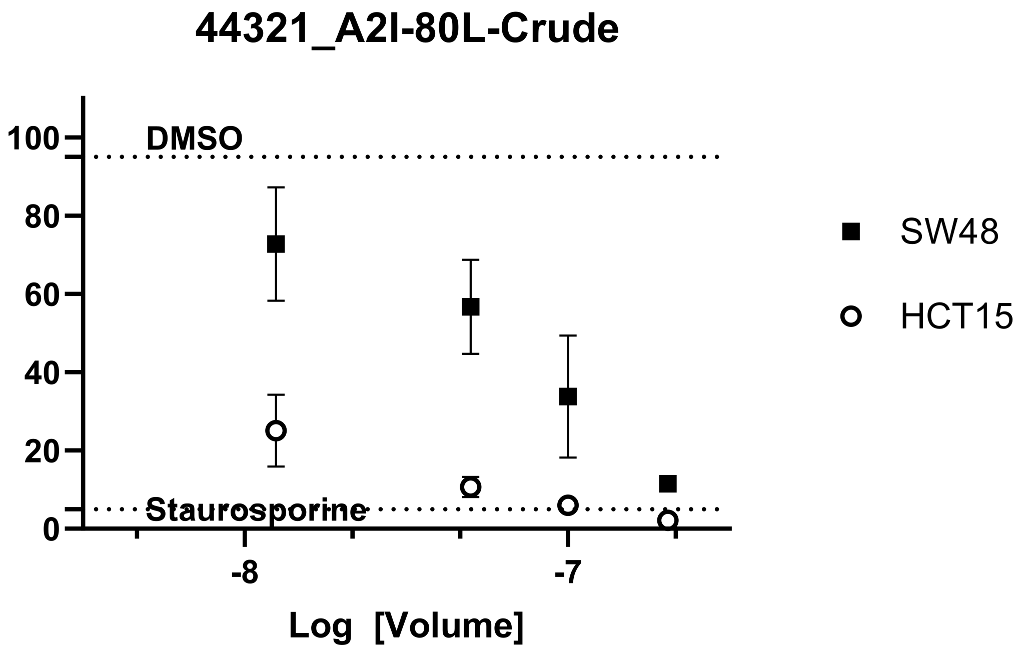

Supplementary Figure 22: Activity of crude extracts generated from 2-aminobenzamide-actiphenol producing strain *Streptomyces actiphen* against SW48 (CCL231, Colon Cancer) and HCT15 (CCL-225 Colon Cancer) cell line. Error bars represent the standard deviation.

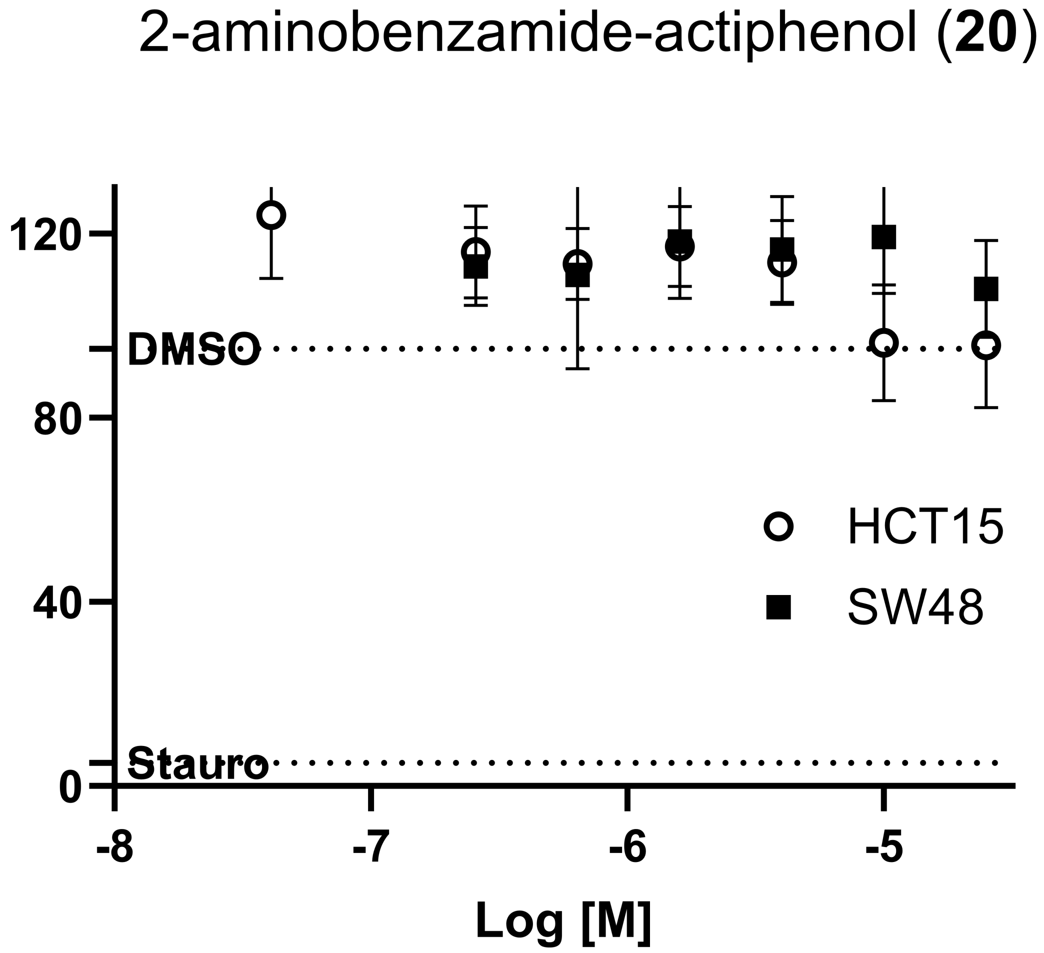

Supplementary Figure 23: Activity of the isolated novel molecule 2-aminobenzamide-actiphenol against SW48 (CCL231, Colon Cancer) and HCT15 (CCL-225 Colon Cancer) cell line. Error bars represent the standard deviation.

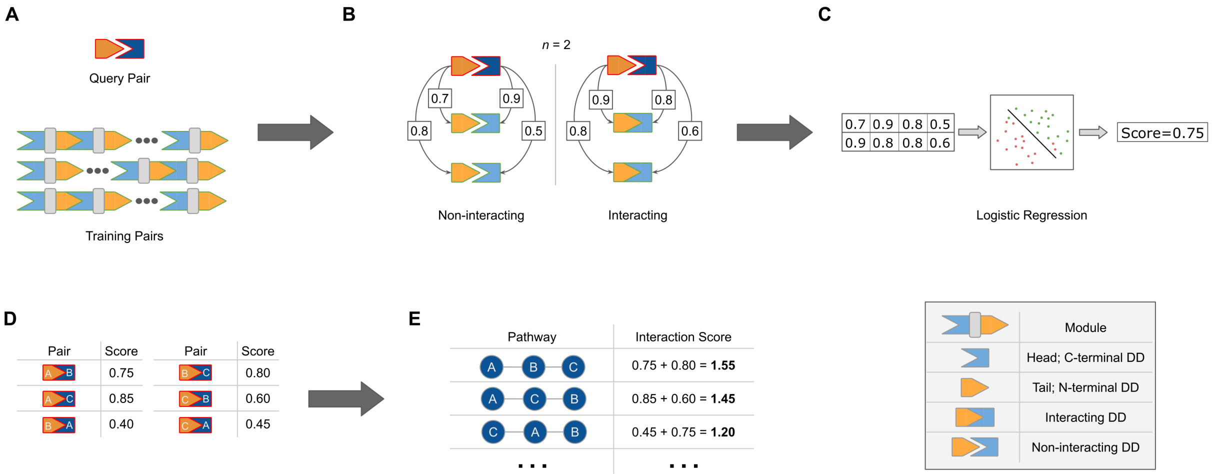

Supplementary Figure 24: Overview of the PNN algorithm. Given (A) query and training head-tail pairs, (B) each query head(tail) docking domain is blasted against all the training head(tail) docking domains, and k top-scoring matches are selected ($k = 3$). Then, (C) k^2 pairs are formed between them ($k = 3$), and eight features are computed between these pairs. These features include the number of interacting / non-interacting neighbors, the sum of the head BLAST bit-score of interacting / non-interacting neighbors, the sum of the tail BLAST bit-score of interacting / non-interacting neighbors, the sum of the head and tail BLAST bit-score of interacting / non-interacting neighbors. (D) Based on these eight features, a random forest classifier is trained to predict whether each query pair is interacting or not. (E) The overall score of each candidate pathway is computed as the sum of the interaction score and continuity score, where the interaction score is the sum of the interaction score for all the adjacent pairs in the pathway, and the continuity score is w times the number of adjacent pairs in the pathway that are also adjacent in the BGC. Then the pathways are ranked based on their overall scores, and the top five ranking pathways are retained.

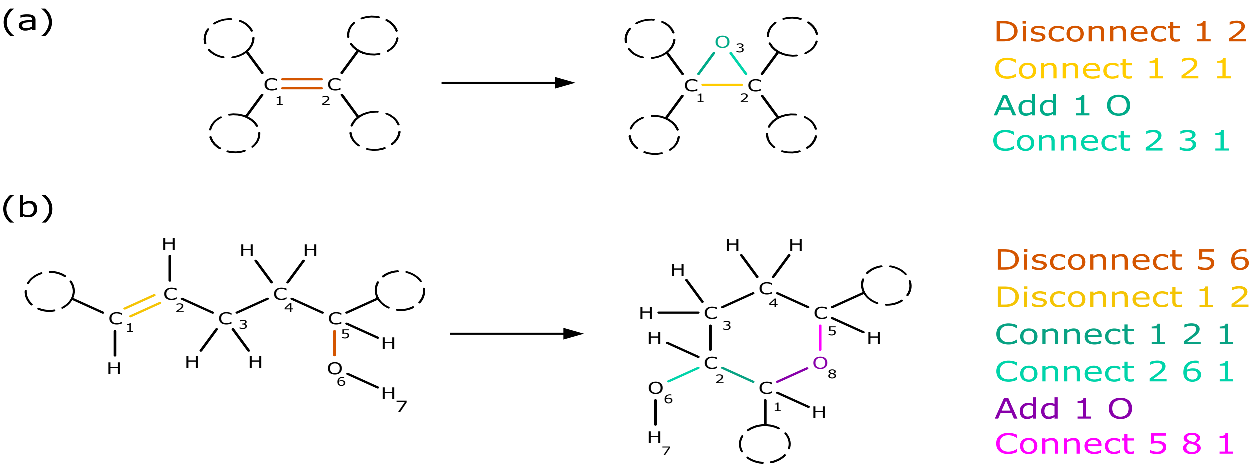

Supplementary Figure 25: Enzymatic modifications of polyketides are expressed in a computer-readable format for (a) epoxidation enzyme (e.g. in Abyssomicin C) [1], and (b) pyran ring formation (e.g. in Ambruticin) [2]. In our format, we use commands disconnect/connect (for bonds) and add/remove (for chemical substructures). For example, in part (a), "disconnect 1 2" removes the double bond between carbon atoms with index 1 and 2, while "add 1 O" adds an oxygen atom and connects it with a carbon atom with index 1. Moreover, "connect 2 3 1" connects this added oxygen (which automatically gets index 3) to the carbon with index 2.

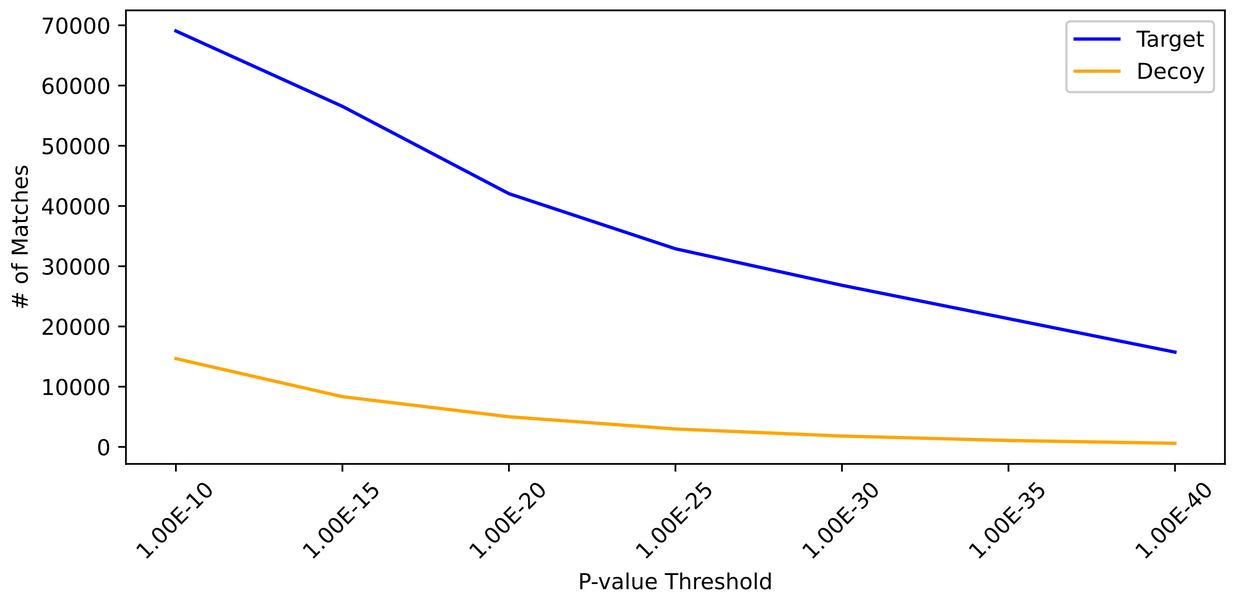

Supplementary Figure 26: Number of polyketide-spectrum matches/peptides identified by Seq2PKS at different p-value thresholds for the target and decoy databases for nystatin. The X-axis represents different p-value thresholds, and the Y-axis represents the number of identified polyketide-spectrum matches or peptides.

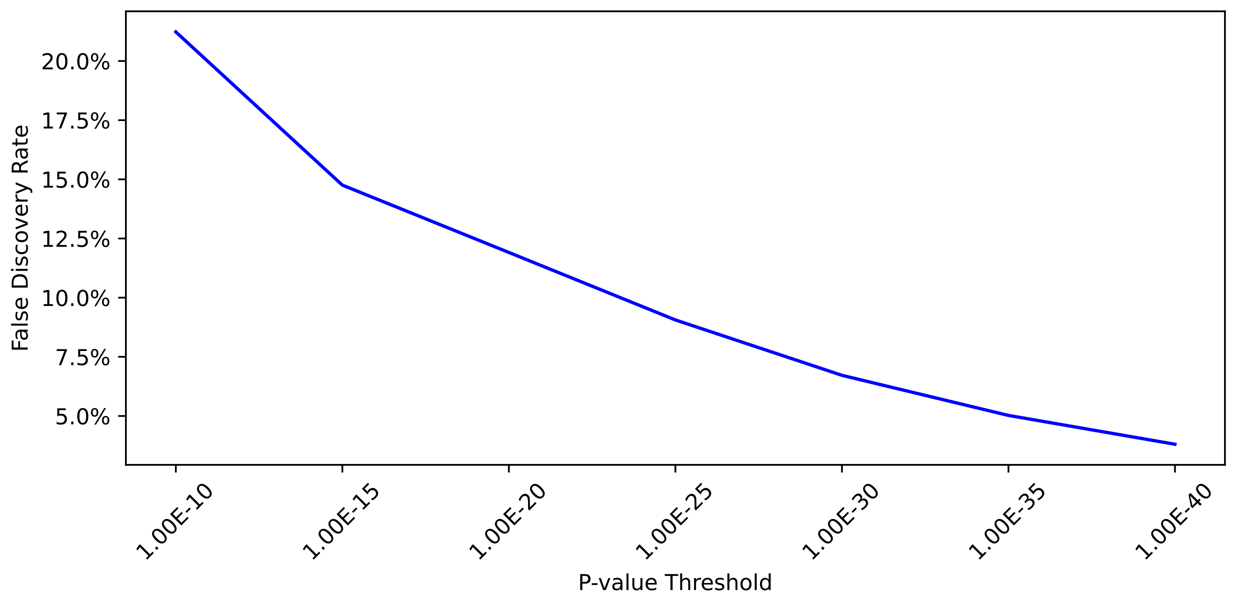

Supplementary Figure 27: False discovery rate of Seq2PKS at different p-value thresholds for the target and decoy databases for nystatin. The X-axis represents different p-value thresholds, and the Y-axis represents the corresponding false discovery rate.

Supplementary Table 1: Gene annotation for identified monazomycin BGC in *Streptomyces cinnamoneus* NRRL B-24434

Name	Location	Nucleotide	Amino Acid	Hypothesized Function
Contig 1	2 - 1,417	1,416	472	LuxR family transcriptional regulator
Contig 2	1,573 - 2,337	765	254	alpha/beta fold hydrolase (thioesterase)
Contig 3	3,173 - 5,959	2,787	928	LuxR family transcriptional regulator
Contig 4	6,241 - 7,899	1,659	552	NAD(P)/FAD-dependent oxidoreductase
Contig 5	8,450 - 9,154	705	234	thioesterase
Contig 6	9,280 - 10,692	1,413	470	class I adenylate-forming enzyme family
Contig 7	10,751 - 11,701	951	316	ACP S-malonyltransferase
Contig 8	11,733 - 13,025	1,293	430	histidine kinase
Contig 9	13,359 - 14,900	1,542	513	glycosyltransferase
Contig 10	14,973 - 16,214	1,242	413	cytochrome P450
Contig 11	16,343 - 19,726	3,384	1,127	SDR family NAD(P)-dependent oxidoreductase
Contig 12	20,001 - 29,660	9,660	3,219	type I polyketide synthase
Contig 13	29,748 - 45,353	15,606	5,201	type I polyketide synthase
Contig 14	45,420 - 56,489	11,070	3,689	type I polyketide synthase
Contig 15	56,536 - 71,967	15,432	5,143	type I polyketide synthase
Contig 16	72,473 - 86,383	13,911	4,636	type I polyketide synthase
Contig 17	86,428 - 107,196	20,769	6,922	type I polyketide synthase
Contig 18	107,219 - 125,716	18,498	6,165	type I polyketide synthase
Contig 19	126,193 - 138,252	12,060	4,019	type I polyketide synthase
Contig 20	138,360 - 138,524	165	55	unknown

Supplementary Table 2: Gene annotation for 2-aminobenzamide-actiphenol BGC.

Name	Location	Nucleotide	Amino Acid	Hypothesized Function
Contig 1	2 - 901	900	299	polar amino acid ABC transporter
Contig 2	905 - 1,657	753	250	ABC transporter
Contig 3	1,753 - 2,538	786	261	unknown
Contig 4	2,608 - 3,297	690	229	GntR family transcriptional regulator
Contig 5	3,343 - 4,149	807	268	short-chain dehydrogenase/reductase SDR
Contig 6	4,370 - 5,017	648	215	unknown
Contig 7	5,017 - 5,667	651	216	unknown
Contig 8	5,664 - 6,539	876	291	ABC transporter
Contig 9	6,536 - 7,639	1,104	367	transport system permease protein
Contig 10	7,620 - 8,774	1,155	384	iron compound ABC transporter
Contig 11	9,012 - 9,158	147	48	unknown
Contig 12	9,460 - 10,227	768	255	unknown
Contig 13	10,475 - 11,200	726	241	unknown
Contig 14	11,658 - 12,803	1,146	381	unknown
Contig 15	12,858 - 13,307	450	149	unknown
Contig 16	14,117 - 14,467	351	116	unknown
Contig 17	14,738 - 15,448	711	236	unknown
Contig 18	16,234 - 16,437	204	67	unknown
Contig 19	16,458 - 17,060	603	200	unknown
Contig 20	17,162 - 17,626	465	154	unknown
Contig 21	17,662 - 18,789	1,128	375	serine/threonine protein kinase
Contig 22	18,966 - 19,784	819	272	unknown
Contig 23	20,001 - 40,511	20,511	6,836	type I polyketide synthase
Contig 24	40,604 - 42,643	2,040	679	asparagine synthase
Contig 25	42,660 - 42,917	258	85	putative acyl carrier protein
Contig 26	42,921 - 43,796	876	291	type I polyketide synthase
Contig 27	44,081 - 47,410	3,330	1,109	transcriptional regulator
Contig 28	47,582 - 50,980	3,399	1,132	NRP synthase
Contig 29	51,124 - 52,323	1,200	399	cytochrome P450
Contig 30	52,477 - 53,262	786	261	short-chain dehydrogenase/reductase SDR
Contig 31	53,900 - 54,988	1,089	362	flavin oxidoreductase
Contig 32	55,091 - 56,020	930	309	LysR family transcriptional regulator

Supplementary Table 3: NMR result for 2-aminobenzamide-actiphenol.

Position	δ_C (type)	δ_H, multiplets (J in Hz)	COSY	HMBC	ROESY
1	159.3 (C)				
1-OH		12.33, s		1, 2, 6	
2	127.0 (C)				
3	136.9 (CH)	7.45, s		1, 5, 14, 15	14, 15a, 15b
4	130.0 (C)				
5	127.6 (CH)	7.82, s		1, 3, 7, 15	8a, 8b, 15a, 15b
6	118.7 (C)				
7	205.6 (C)				
8a	42.9 (CH_2)	3.18, d (6.4)	9	7, 9, 10, 13	5, 10a, 10b, 13a, 13b
8b	42.9 (CH_2)	3.18, d (6.4)	9	7, 9, 10, 13	5, 10a, 10b, 13a, 13b
9	26.6 (CH)	2.62, m	8a, 8b		
10a	37.5 (CH_2)	2.39, dd (16.5, 10.7)	10b	11, 12	5
10b	37.5 (CH_2)	2.58, dd (16.5, 10.7)	10a	11, 12	5
11	173.4 (C)				
11-NH		10.76, s		10, 13	
12	173.4 (C)				
13a	37.5 (CH_2)	2.39, d (16.5, 10.7)	13b	11, 12	5
13b	37.5 (CH_2)	2.58, d (16.5, 10.7)	13a	11, 12	5
14	15.8 (CH_3)	2.17, s		1, 2, 3	3
15a	45.9 (CH_2)	4.30, s		3, 4, 5, 16	3, 5, 17
15b	45.9 (CH_2)	4.30, s		3, 4, 5, 16	3, 5, 17
15-NH		8.47, br s			
16	149.9 (C)				
17	112.1 (CH)	6.69, d (8.4)	18	19, 21	15a, 15b, 18
18	132.8 (CH)	7.23, t (7.9)	17, 19	16, 20	17, 19
19	114.9 (CH)	6.54, t (7.5)	18, 20	17, 21	18, 20
20	129.5 (CH)	7.61, d (7.8)	19	16, 18, 22	18, 19
21	114.9 (C)				
22	171.9 (C)				
22-NHa		7.17, br s			
22-NHb		7.86, br s			

References

[1] Elvira Gottardi, Joanna Krawczyk, Hanna Suchodoletz, Simone Schadt, Agnes Mühlenweg, Gabriel Uguru, Stefan Pelzer, Hans-Peter Fiedler, Mervyn Bibb, James Stach, Roderich Süssmuth. *Abyssomicin Biosynthesis: Formation of an Unusual Polyketide, Antibiotic-Feeding Studies and Genetic Analysis. Chembiochem*, 2011, 12: 1401-1410. https://doi.org/10.1002/cbic.201100172

[2] Gary Marshall, G Broadhead, BK Leskiw, GD Wright. *D-Ala-D-Ala ligases from glycopeptide antibiotic-producing organisms are highly homologous to the enterococcal vancomycin-resistance ligases VanA and VanB. Proceedings of the National Academy of Sciences of the United States of America*, 1997, 94: 6480-6483. https://doi.org/10.1073/pnas.94.12.6480
